# ATOX1 alleviates radiation‐induced cardiac injury by modulating AMPK/NRF2 to inhibit myocardial oxidative stress and mitochondrial dysfunction

**DOI:** 10.1002/ccs3.70079

**Published:** 2026-05-15

**Authors:** Wen Deng, Li Su

**Affiliations:** ^1^ Department of Radiation Oncology The First Affiliated Hospital of Kunming Medical University Kunming Yunnan China; ^2^ Second Department of Cardiology The First Affiliated Hospital of Kunming Medical University Kunming Yunnan China

**Keywords:** AMPK/NRF2 signaling pathway, ATOX1, mitochondrial dysfunction, oxidative stress, radiation‐induced heart disease

## Abstract

Radiation‐induced heart disease (RIHD) is a myocardial lesion caused by radiation exposure, and its pathogenesis is closely associated with oxidative stress. ATOX1 has been demonstrated to regulate oxidative stress, but its mechanism in RIHD remains unclear. We analyzed ATOX1 expression (Western blot [WB], RT‐PCR), cardiomyocyte proliferation (MTT, IF), apoptosis (TUNEL), AMPK signaling (WB, IF), and mitochondrial function (reactive oxygen species [ROS], mPTP, JC‐1) in vitro. A thoracic irradiation model was used in cardiomyocyte‐specific ATOX1 knockout mice. Tissue analysis included IHC for ATOX1, KI‐67, p‐AMPK, and assessment of myocardial injury (ELISA, RT‐PCR, and Masson's staining). Irradiation significantly reduced cardiomyocyte proliferation and increased apoptosis. ATOX1 levels plummeted in irradiated cardiomyocytes, accompanied by mitochondrial ROS surges and disrupted integrity. Irradiation suppressed the AMPK/NRF2 axis, an effect reversed by ATOX1 overexpression. In mice, ATOX1 knockout exacerbated radiation‐induced myocardial tissue damage. ATOX1 mitigates irradiation‐induced cardiac damage by promoting mitochondrial and redox homeostasis in cardiomyocytes through AMPK/NRF2 pathway activation.

## Graphical abstract

This study found that ATOX1 can alleviate radiation‐induced oxidative stress and mitochondrial damage, maintain mitochondrial function, and reduce oxidative stress by activating the AMPK/NRF2 signaling pathway, ultimately exerting a cardioprotective effect. This study provides a new target for the prevention and treatment of radiation‐induced heart damage.

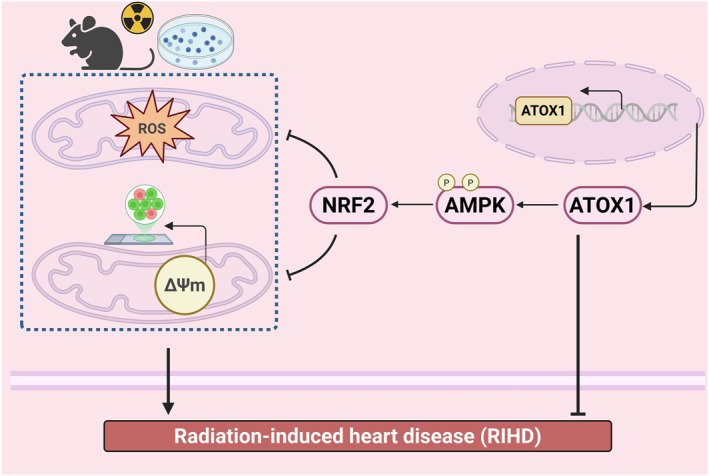

## 1 INTRODUCTION

Radiotherapy is a cornerstone treatment for thoracic malignancies, including breast cancer, esophageal cancer, and lung cancer. However, even with enhanced precision, it inevitably exposes the heart to radiation. This exposure causes radiation‐induced heart disease (RIHD), which encompasses a spectrum of cardiac disorders—including pericarditis, cardiomyopathy, and coronary artery disease^1^—which results in a degraded quality of life in those patients. RIHD also markedly elevates cardiac‐related mortality post‐treatment. For instance, lung cancer patients undergoing radiotherapy face a markedly higher risk of cardiac death compared to those who do not.^2^ Improving patient outcomes in RIHD hinges on a deeper understanding of its pathological mechanisms. The condition is characterized by a range of pathological changes, including inflammation, oxidative stress, and damage to mitochondria and the endoplasmic reticulum. Oxidative stress and mitochondrial dysfunction are particularly prominent.^1^ However, the molecular mechanisms driving these changes remain unclear.

Oxidative stress is a critical factor in RIHD pathogenesis. In breast cancer, higher ratios of oxidative stress markers are strongly associated with a greater long‐term risk of cardiovascular disease induced by radiotherapy.^3^ In a healthy heart, reactive oxygen species (ROS) signaling helps regulate critical processes, including calcium handling, excitation‐contraction coupling, cardiomyocyte maturation, cardiac development, and vascular tone. However, after irradiation, cardiomyocytes churn out excessive ROS, which can mess with the function of biomolecules, such as proteins, DNA, and lipids, causing a cascade of pathological changes.^4^ Oxidative stress during RIHD is often accompanied by mitochondrial dysfunction.^5^ This dysfunction usually involves impaired energy metabolism due to a combination of factors: repression of the respiratory chain, damage to the mitochondrial membrane, lowered enzyme activity, and damage to mtDNA. These issues set off a chain reaction of damage.^6^ γ‐ray exposure has been found to induce oxidative stress and mitochondrial damage in iPSC‐CMs, marked by increased mitochondrial permeability transition pore (mPTP) opening, decreased membrane potential, and higher ROS levels.^7^ This discovery underscores the importance of oxidative stress in RIHD and its close association with mitochondrial dysfunction, hinting at a potential target for therapeutic interventions.

Cells can keep oxidative stress in check by modulating various signaling pathways, with the AMPK/NRF2 pathway being a key player. Its activation can curb doxorubicin‐induced oxidative stress in cardiomyocytes and reduce the risk of cardiomyopathy.^8^ AMPK and NRF2 are key regulators within cells, ensuring that energy and redox balance are maintained to support normal cellular function. AMPK acts as an energy sensor inside cells that monitors the AMP/ATP ratio to gauge energy status. AMPK is key for maintaining energy balance and supporting cell growth, and it also helps cells respond to oxidative stress and inflammation.^9^ NRF2 is a critical cellular factor that binds to antioxidant response elements (AREs) and activates the expression of enzymes that work in concert to neutralize free radicals and detoxify harmful substances.^9^ AMPK can bolster the resilience of cardiomyocytes to oxidative stress and curb ischemia‐reperfusion injury by phosphorylating NRF2 or its upstream regulators.^10^ ATOX1 is a key regulator of mitochondrial antioxidant capacity and cellular oxidative stress.^11^ However, the detailed molecular mechanisms underlying the antioxidant functions of ATOX1 are still not fully understood. Our study explored whether ATOX1 can mitigate radiation‐induced cardiac damage by modulating the AMPK/NRF2 signaling pathway, which is critical for maintaining myocardial redox homeostasis and mitochondrial integrity.

RIHD remains a critical issue following thoracic radiotherapy, driven largely by oxidative stress and mitochondrial dysfunction. Despite these insights, the underlying molecular mechanisms are still unclear. ATOX1 is a potential regulator of cellular antioxidant responses. Our studies demonstrate that ATOX1 can activate the AMPK/NRF2 pathway, which helps protect the heart from oxidative stress and mitochondrial damage caused by radiation. This work aims to elucidate the cardioprotective role of ATOX1 in RIHD and its potential as a therapeutic strategy.

## 2 MATERIALS AND METHODS

### 2.1 Cell culture

The rat cardiomyocyte cell line H9C2 (BNCC337726) was obtained from BNCC (China). These cells were maintained in DMEM‐H medium supplemented with 10% fetal bovine serum (FBS) and 1% penicillin‐streptomycin. Cultures were incubated at 37°C in a humidified atmosphere containing 5% CO_2_ using an incubator from Thermo Fisher (USA). All cell culture reagents, including DMEM‐H, FBS, and penicillin‐streptomycin, were sourced from Gibco (USA). Cells were exposed to 16 Gy of X‐irradiation prior to analysis.

Mouse primary ventricular cardiomyocytes (M‐VCM) were purchased from Cellverse (China) and cultured using the company's dedicated culture medium. Prior to the start of the experiment, relevant tests were conducted using 16 Gy X‐ray irradiation. All cell experiments were strictly conducted according to the standards of randomization and blinding, with random sampling, random drug addition, hidden grouping, and objective analysis of data.

### 2.2 Cell transfection

sh‐NC and sh‐AMPK lentiviral vectors, pcDNA3.1 empty vector (oe‐NC) and the pcDNA3.1‐ATOX1 overexpression plasmid (oe‐ATOX1) were provided by Genepharma (China). For lentiviral infection, the lentiviral particles were used to directly infect cells. Plasmid transfection was carried out using Lipofectamine 3000 (Invitrogen, 3000015, USA). H9C2 cells were harvested at 80% confluence and switched to serum‐free medium. In a 1.5 mL centrifuge tube, 125 μL of serum‐free medium and 3.75 μL of Lipofectamine 3000 reagent were mixed. In another 1.5 mL centrifuge tube, 125 μL of serum‐free medium, 5 μL of P3000™ reagent, and 2.5 μg of plasmid were combined. The plasmid mixture was added to the first tube and mixed well. After a 10–15 min incubation, the mixture was added to the cells and incubated at 37°C. After 4 h, the medium was replaced with serum‐containing medium, and the cells were cultured for an additional 48 h before mRNA expression levels were measured.

### 2.3 RNA extraction and RT‐PCR

To extract RNA from cells and tissues, 1 mL of Trizol reagent (Solarbio, R1100, China) was added to each well, and the mixture was transferred to 1.5 mL centrifuge tubes. Each tube was mixed with 200 μL of chloroform and vortexed thoroughly, and then centrifuged at 4°C to separate RNA from proteins and other impurities. The aqueous phase was mixed with an equal volume of isopropanol (Macklin, China) and left at room temperature for 10 min before centrifugation to collect the RNA pellet. The pellet was washed with 1 mL of 75% ethanol, centrifuged to remove the supernatant, and air‐dried. The RNA pellet was dissolved in DEPC water (Sangon Biotech, China), and its concentration was measured using a Nanodrop spectrophotometer (Thermo Fisher, USA). The RNA was then reverse‐transcribed into cDNA using the HiScript IV All‐in‐One Ultra RT SuperMix for RT‐PCR (Vazyme, R433‐01, China) and stored at −20°C.

The reaction mixture was prepared following the manufacturer's protocol. Taq Pro Universal SYBR qPCR Master Mix (Vazyme, Q712‐02/03, China) was used as the nucleic acid dye, and β‐actin was used as the reference gene. RT‐PCR reactions and detection were carried out on the Applied Biosystems® 7500 Fast Real‐Time PCR System (Thermo Fisher, USA). Relative gene expression was analyzed using the 2^−ΔΔCt^ method, and the primer information is provided in Table 1.

**TABLE 1:** Primers' information.

Genes	Primers (5′–3′)
*β‐actin* (rat)‐F	CACTGTCCACCTTCCAGCAG
*β‐actin* (rat)‐R	GAAAGGGTGTAAAACGCAGCTC
*β‐actin* (mouse)‐F	GTACTCTGTGTGGATCGGTGG
*β‐actin* (mouse)‐R	GCAGCTCAGTAACAGTCCG
Atox1 (rat)‐F	GACATCCTGCTGGCAACTCT
Atox1 (rat)‐R	ACCAGATCAACAGTCTGCCTC
Col3a1 (mouse)‐F	ACGTAAGCACTGGTGGACAG
Col3a1 (mouse)‐R	CAGGAGGGCCATAGCTGAAC
Col1a1 (mouse)‐F	AGCACGTCTGGTTTGGAGAG
Col1a1 (mouse)‐R	GACATTAGGCGCAGGAAGGT
Tgfβ (mouse)‐F	ACCGGACAGTAGAGAGGGAC
Tgfβ (mouse)‐R	CTCCCCACCTGCGAAGAAAT

### 2.4 Western blot (WB)

Total protein was extracted from cells using a total protein extraction kit (Solarbio, BC3710, China). The samples were then separated by SDS‐PAGE and transferred onto a PVDF membrane (MilliporeSigma, USA). After blocking with skim milk, the membrane was incubated with primary antibodies overnight at 4°C. The primary antibodies included anti‐ATOX1 (ABclonal, A6874, China), anti‐p‐AMPK (ABclonal, AP1002, China), anti‐AMPK (ABclonal, A1229, China), anti‐NRF2 (ABclonal, A1244, China), anti‐HO‐1 (ABclonal, A19062, China), anti‐β‐Actin (ABclonal, AC038, China), and antihistone H3 (Proteintech, China). Following extensive washing, the membrane was incubated with horseradish peroxidase (HRP)‐conjugated secondary antibodies (goat anti‐rabbit IgG, Beyotime, A0208, China; goat anti‐mouse IgG, Beyotime, A0216, China) for 2 h at room temperature. Protein bands were visualized using a Super Sensitive ECL Chemiluminescent Substrate (Beyotime, P001S) and captured with a ChemiScope 6200 Image Analyzer (Clinx, China).

### 2.5 MTT assay

The proliferation of H9C2 cells was evaluated using an MTT assay kit (Solarbio, M1020, China). Cells were seeded in a 96‐well plate at a density of 5000 cells per well. Once the cells had adhered, they were treated as specified. The cells were then incubated with culture medium containing 10% MTT solution for 4 h. The medium was removed, and 110 μL of formazan dissolution solution was added to each well. The plate was shaken gently on a shaker for 10 min to dissolve the formazan crystals. The absorbance at 490 nm was measured using a microplate reader (Thermo Fisher, USA).

### 2.6 Immunofluorescence (IF)

To prepare mouse heart tissue sections, fresh heart tissue was immersed in 4% paraformaldehyde (Beyotime, P0099, China) for 24 h. The tissue was trimmed to an appropriate size and embedded in paraffin. After the paraffin solidified, the tissue was cut into 5‐μm sections and mounted on slides by drying. The sections were dewaxed and rehydrated using xylene and graded ethanol. Antigen retrieval was performed using EDTA antigen retrieval solution, followed by rinsing with PBS. Excess moisture was removed from the sections, which were then treated with 3% H_2_O_2_ at room temperature for 10 min to quench endogenous peroxidase activity. After three rinses with PBS, the sections were blocked with 3% BSA for 30 min and incubated with primary antibodies at 37°C. Cells in 6‐well plates were fixed with 75% ethanol, permeabilized with 0.25% Triton X‐100 (Beyotime, P0096‐100 mL, China), and blocked with 3% BSA in PBS. The cells were then incubated with primary antibodies at 4°C.

The primary antibodies used were anti‐Ki‐67 (Proteintech, 84432‐1‐RR, China) and anti‐p‐AMPK (Thermo Fisher, 44‐1150G, USA). These antibodies were incubated with the tissue/cells for 1 h at room temperature. The tissue/cells were then treated with the secondary antibody, Goat anti‐Rabbit IgG (Heavy chain) Superclonal™ Secondary Antibody, Alexa Fluor™ 488 (Thermo Fisher, A27034, USA), for 30 min at room temperature. DAPI staining solution (Beyotime, C1006, China) was applied, and the fluorescence signals were observed under a fluorescence microscope (KEYENCE, Japan).

### 2.7 TUNEL

To quantify DNA damage, we used the One‐Step TUNEL Apoptosis Detection Kit (Beyotime, C1089, China) to detect DNA fragmentation in cells from different experimental groups. After completing the experimental treatments, cells were washed three times with PBS, resuspended, and harvested. Cells were fixed with 4% paraformaldehyde for 30 min and permeabilized with PBS containing 0.3% Triton X‐100 at room temperature for 5 min. Cells were then incubated with the TUNEL reaction buffer in the dark at 37°C for 60 min. Fluorescence signals were visualized using a fluorescence microscope (KEYENCE, Japan) after washing the cells three times with PBS.

### 2.8 JC‐1 staining

To assess mitochondrial membrane potential, we used the JC‐1 Mitochondrial Membrane Potential Assay Kit (Abcam, ab113850, UK). Cells were seeded in a 96‐well plate at a density of 1.5 × 10^4^ cells per well and cultured overnight. After washing the cells twice with PBS, 100 μL of JC‐1 working solution was added to each well. The plate was incubated in the dark at 37°C for 10 min, and then washed twice with buffer. Fluorescence was observed and analyzed using a fluorescence microscope (KEYENCE, Japan).

### 2.9 Mitochondrial ROS assessment

We used MitoSOX™ Red (Thermo Fisher, M36008, USA) to measure mitochondrial ROS. Cells were treated with 1 mM staurosporine (MedChemExpress, HY‐15141, USA) to induce apoptosis. Then, 30 mM MitoPQ was added to low‐glucose medium and incubated overnight to induce mitochondrial superoxide production. Fluorescence was visualized and analyzed using a fluorescence microscope (KEYENCE, Japan).

### 2.10 Mitochondrial permeability transition analysis

To assess mPTP opening in cardiomyocytes, we used the in vivo mPTP fluorescence detection kit (Beyotime, C2009S, China). Cells were first washed with PBS. Then, 250 μL of Calcein AM staining solution and fluorescence quenching working solution were added to each well of a 24‐well plate. The plate was gently agitated to ensure uniform dye coverage over all cells and incubated in the dark at 37°C for 30 min. After incubation, the medium was replaced with fresh, pre‐warmed (37°C) culture medium and incubated again in the dark at 37°C for another 30 min. The medium was then removed, and the cells were washed 2–3 times with PBS. The detection buffer was added, and green fluorescence was observed using a fluorescence microscope (KEYENCE, Japan).

### 2.11 Animal experiment

Adult C57BL/6 mice (6 weeks old, 16 ± 2 g) were acquired from Hangzhou Ziyuan Experimental Animal Technology Co., Ltd, and groups were randomly divided (*n* = 6). For the generation of cardiac‐specific ATOX1 conditional knockout (CKO‐ATOX1) mice, we performed breeding between ATOX1 flox homozygous (fl/fl) mice and α‐myosin heavy chain‐MerCreMer (αMHC‐MCM) mice.

To irradiate the thoracic region of the mice, we used the X‐RAD 225XL biological irradiator (Rad Source Technologies, USA) to deliver a 20 Gy X‐ray dose at 100 cGy/min. The rest of the body was shielded with lead to protect against radiation. After irradiation, the weight changes of mice were monitored and recorded every 5 days by uninformed personnel for statistical analysis. After 35 days, the mice were euthanized, and we weighed their hearts and collected heart tissue and serum samples. All animal research protocols were approved by the Animal Ethics Committee of Guangdong Medical Laboratory Animal Center, approval number D202506‐11.

### 2.12 Immunohistochemistry (IHC)

Heart tissue sections were processed for immunohistochemistry. Briefly, paraffin‐embedded sections were dewaxed in xylene and rehydrated through graded ethanol. Antigen retrieval was performed using EDTA buffer, followed by thorough rinsing with PBS. Sections were treated with 3% H_2_O_2_ for 10 min at room temperature to block endogenous peroxidase activity. After rinsing, sections were blocked with 3% BSA for 30 min to prevent nonspecific binding. The primary antibody, anti‐ATOX1 (Proteintech, 31090‐1‐AP, China), was applied and incubated overnight at 4°C. Following three PBS rinses, the secondary antibody, Goat anti‐Rabbit IgG H&L (HRP) (Abcam, ab6721, UK), was applied and incubated at 37°C for 30 min. Sections were then stained with DAB (Solarbio, DA1015, China) and counterstained with hematoxylin (Solarbio, G1120, China). Each step was followed by thorough rinsing with water. Finally, sections were dehydrated, cleared, and mounted with neutral resin. Slides were examined under an optical microscope (Mshot, China).

### 2.13 ELISA

To assess myocardial injury, we measured the levels of CKMB, cTnT, and NT‐proBNP in mouse serum using ELISA kits. Specifically, the Mouse CKMB (Creatine Kinase MB Isoenzyme) ELISA Kit (Elabscience, E‐EL‐M0355, China), Mouse cTnT/TNNT2 (Troponin T Type 2, Cardiac) ELISA Kit (Elabscience, E‐EL‐M1801, China), and Mouse NT‐proBNP (N‐terminal pro‐Brain Natriuretic Peptide) ELISA Kit (Elabscience, E‐EL‐M0834, China) were employed. Blood samples were processed to extract serum, which was then added to the microplate wells (100 μL per well) and incubated at 37°C for 90 min. Biotinylated antibody solution (100 μL) was added next and incubated for 1 h. After washing with PBS, HRP solution (100 μL) was added and incubated for 30 min. TMB substrate was then added and incubated in the dark for 15 min. The reaction was stopped with 50 μL of stop solution, and absorbance was measured at 450 nm using a microplate reader (Thermo Fisher, USA).

### 2.14 Masson staining

Mouse heart tissue sections were stained with Masson's trichrome staining solution (Servicebio, G1006, China). The sections were deplasticized and hydrated as per the manufacturer's protocol. They were then soaked in Masson Solution A overnight. Next, the sections were heated in Masson Solution A at 37°C for 30 min. After a brief rinse in running water for 30 s, Masson Solutions B and C were mixed in equal amounts just before use. The sections were stained in this mixture for 1 min and then rinsed under running water. They were differentiated in 1% hydrochloric acid alcohol for about 1 min, stained in Masson Solution D for 6 min, and treated in Masson Solution E for about 1 min without rinsing. Excess Masson Solution E was drained off, and the sections were directly immersed in Masson Solution F for 10 s. The sections were then differentiated in 1% glacial acetic acid, dehydrated in anhydrous ethanol, cleared in butanol and xylene, and mounted with neutral resin. The slides were observed under an optical microscope (Mshot, China).

### 2.15 Statistical analysis

All in vitro experiments were conducted with three independent replicates. Results are presented as mean ± standard deviation. GraphPad Prism 8.0 (GraphPad, USA) was used to process the data and generate the figures. Student *t*‐test was used to compare the differences between two groups; one‐way ANOVA was used to analyze differences between multiple groups, and Dunnett's test was used for pairwise comparisons to avoid false positives. A *p*‐value of less than 0.05 was considered statistically significant.

## 3 RESULTS

### 3.1 ATOX1 encourages the survival of cardiomyocytes after radiation exposure

**FIGURE 1:**
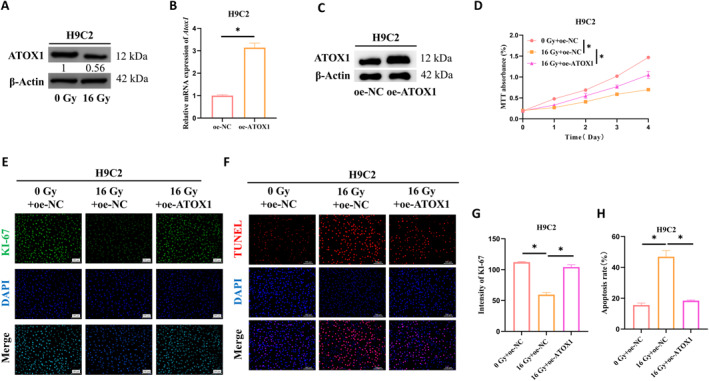
ATOX1 encourages the survival of cardiomyocytes after radiation exposure. (A) WB analysis of ATOX1 protein expression changes in H9C2 cells following radiation; (B, C) RT‐PCR and WB assessed ATOX1 overexpression efficiency; (D) MTT assay of H9C2 cell proliferation; (E, G) IF analysis of KI‐67 expression levels in H9C2 cells; (F, H) TUNEL assay of apoptosis in H9C2 cells. *p < 0.05. WB, western blot.

We initially examined the role of ATOX1 in cardiomyocytes subjected to radiation. After exposing cardiomyocytes to 16 Gy of radiation, we detected a decrease in ATOX1 protein expression (Figure 1A). We then established a cardiomyocyte cell line overexpressing ATOX1 and validated the overexpression efficiency using RT‐PCR and WB (Figure 1B,C). In a rescue experiment with 16 Gy radiation, we compared a control group (0 Gy + oe‐NC), a radiation group (16 Gy + oe‐NC), and an ATOX1‐overexpressing group (16 Gy + oe‐ATOX1). The MTT assay results highlighted that radiation severely curtailed cell proliferation, but this effect was partially offset by ATOX1 overexpression (Figure 1D). IF revealed that radiation exposure dampened KI‐67 expression, a marker of cell proliferation. However, ATOX1 overexpression partially restored this expression, suggesting it enhanced cell proliferation under irradiation (Figure 1E,G). Additionally, TUNEL assays showed that radiation‐induced apoptosis in cardiomyocytes was evidently mitigated by ATOX1 overexpression (Figure 1F,H). Collectively, these results highlight the role of ATOX1 in promoting cardiomyocyte survival post‐radiation exposure.

### 3.2 ATOX1 attenuates radiation‐induced oxidative stress and mitochondrial dysfunction in cardiomyocytes

**FIGURE 2:**
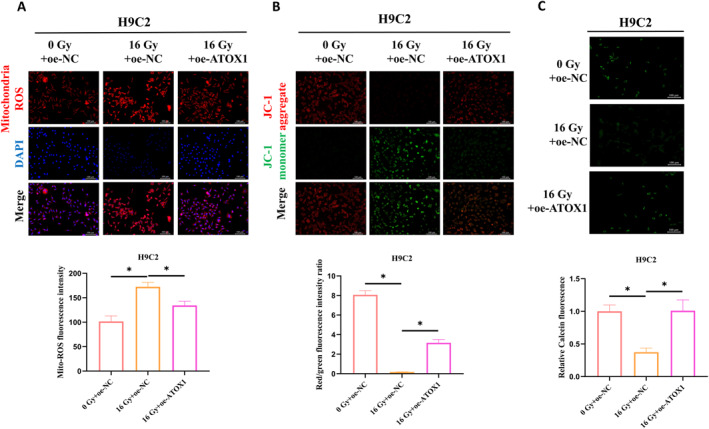
ATOX1 attenuates radiation‐induced oxidative stress and mitochondrial dysfunction in cardiomyocytes. (A) Assay kit analysis of mitochondrial reactive oxygen species levels in H9C2 cells; (B) JC‐1 staining to evaluate changes in mitochondrial membrane potential in H9C2 cells; (C) Assay kit detection of mitochondrial permeability transition pore opening in H9C2 cells. *p < 0.05.

In neuronal cells, ATOX1 mitigates oxidative stress and shields mitochondria from damage.^11^ When mitochondria are compromised, the mPTP opens, leading to a collapse of the membrane potential and higher ROS production, which ultimately triggers apoptosis. We investigated whether ATOX1 could alleviate radiation‐induced oxidative stress and mitochondrial dysfunction in cardiomyocytes by measuring mitochondrial ROS levels. Figure 2A shows that radiation exposure substantially elevated mitochondrial ROS fluorescence, indicative of increased oxidative stress. Overexpression of ATOX1, however, easily counteracted this increase, reducing ROS levels in irradiated cells. JC‐1 staining further revealed that radiation exposure decreased mitochondrial red fluorescence and increased green fluorescence, suggesting a loss of membrane potential. ATOX1 overexpression partially restored red fluorescence intensity (Figure 2B). To determine the effect of radiation on mPTP opening, we measured green fluorescence intensity in cardiomyocytes. Radiation exposure palpably reduced green fluorescence, suggesting increased mPTP opening. However, ATOX1 overexpression restored green fluorescence, effectively inhibiting excessive mPTP opening (Figure 2C). These findings indicate that ATOX1 mitigates radiation‐induced oxidative stress and mitochondrial damage in cardiomyocytes by lowering ROS levels, stabilizing membrane potential, and modulating mPTP opening.

### 3.3 ATOX1 promotes the AMPK/NRF2 signaling

**FIGURE 3:**
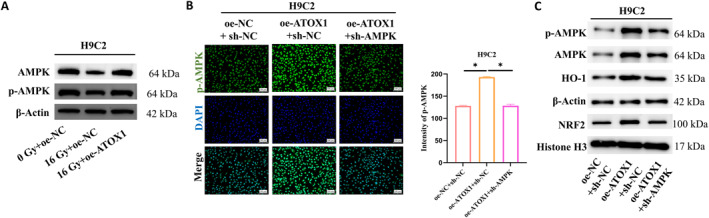
ATOX1 promotes the AMPK/NRF2 signaling. (A) WB analysis of AMPK protein expression and phosphorylation levels following radiation; (B) IF detection of AMPK phosphorylation levels in different cell groups; (C) WB analysis of the activation of the AMPK/NRF2 signaling pathway. *p < 0.05. WB, western blot.

AMPK, functioning as a cellular energy sensor, is indispensable for regulating redox balance and mitochondrial homeostasis. Research studies have shown that activating AMPK can effectively mitigate radiation‐induced skin DNA damage.^12^ Radiation exposure, however, can reduce the levels of phosphorylated AMPK.^13^ To determine whether ATOX1 could modulate the AMPK signaling pathway, we examined the activation of AMPK in cardiomyocytes after radiation exposure at the protein level. The results indicated that radiation exposure reduced AMPK phosphorylation, but this reduction could be reversed by overexpressing ATOX1 (Figure 3A). Based on these observations, we hypothesized that ATOX1 could protect cardiomyocytes from oxidative stress and maintain mitochondrial stability postradiation by activating the AMPK signaling pathway. We used sh‐AMPK lentivirus to infect cardiomyocytes for rescue experiments. IF analysis revealed that overexpressing ATOX1 enhanced the fluorescence intensity of phosphorylated AMPK, which was weakened with the knockdown of AMPK (Figure 3B). We sought to further validate the activation of the AMPK signaling pathway by analyzing key proteins using WB. The data showed that overexpressing ATOX1 potently increased the expression levels of p‐AMPK, HO‐1, and NRF2. However, after the knockdown of AMPK, the expression levels of these proteins returned to levels comparable to the control group, and AMPK protein levels were decreased simultaneously (Figure 3C). These findings suggest that ATOX1 exerts its biological effects by modulating the AMPK/NRF2 signaling pathway. This activation effect may be a crucial mechanism by which ATOX1 combats oxidative stress and protects cells.

### 3.4 ATOX1 alleviates radiation‐induced oxidative stress and mitochondrial dysfunction by activating the AMPK signaling pathway

**FIGURE 4:**
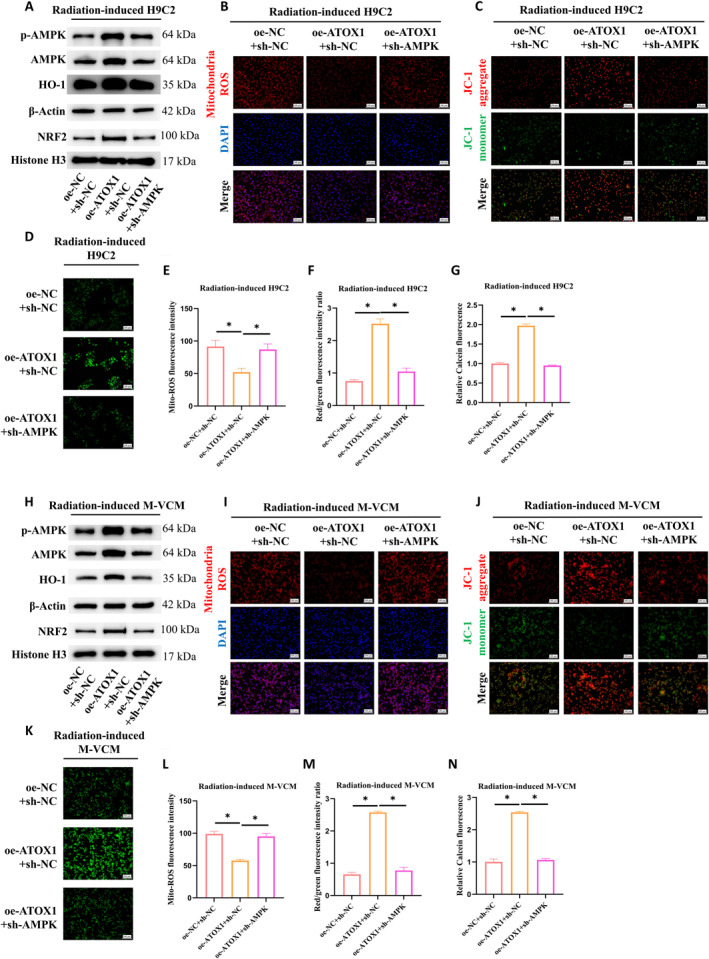
ATOX1 alleviates radiation‐induced oxidative stress and mitochondrial dysfunction by activating the AMPK signaling pathway. (A) WB analysis of the activation status of the AMPK/NRF2 signaling pathway; (B, E) Assay kit measured and quantified mitochondrial ROS levels in H9C2 cells; (C, F) JC‐1 staining evaluated and quantified mitochondrial membrane potential changes in H9C2 cells; (D, G) Assay kit detected and quantified mPTP opening in H9C2 cells. (H) WB assessed the activation of the AMPK/NRF2 signaling pathway. (I, L) Assay kit measured and quantified mitochondrial ROS levels in M‐VCM cells; (J, M) JC‐1 staining evaluated and quantified changes in mitochondrial membrane potential in M‐VCM cells. (K, N) Test kit detected and quantified mPTP opening in M‐VCM cells; *p < 0.05. mPTP, mitochondrial permeability transition pore; ROS, reactive oxygen species; WB, western blot.

To explore the potential of ATOX1 in alleviating radiation‐induced oxidative stress and mitochondrial dysfunction through the AMPK pathway, we established the following experimental setup: a control group receiving DMSO (oe‐NC + sh‐NC), an ATOX1 overexpression group with DMSO (oe‐ATOX1 + sh‐NC), and a group with ATOX1 overexpression combined with AMPK knockdown (oe‐ATOX1 + sh‐AMPK). All groups were exposed to radiation. WB was employed to assess how the AMPK signaling pathway responds to ATOX1 overexpression under radiation stress. The results clearly showed that ATOX1 overexpression robustly activated the AMPK pathway, a response that could be effectively blocked by an AMPK knockdown (Figure 4A). We also examined the impact of these changes on mitochondrial function and oxidative stress in cardiomyocytes. ATOX1 overexpression was found to significantly reduce ROS production in mitochondria. Knocking down AMPK treatment resulted in an increase in ROS fluorescence intensity (Figure 4B,E). In additiont, JC‐1 staining was leveraged to inspect changes in mitochondrial membrane potential. The data unveiled that, in contrast with the control cohort, the ATOX1‐overexpressing group manifested a conspicuously higher count of mitochondrial aggregates, hinting at a more robust mitochondrial membrane potential. However, upon knockdown of AMPK, there was a noticeable transition from aggregates to monomers, which is emblematic of a waning mitochondrial membrane potential and a corresponding dip in mitochondrial functionality (Figure 4C,F). Lastly, mPTP detection spotlighted a more intense green fluorescence in the overexpressing group, a trend that was blunted by AMPK knockdown (Figure 4D,G). Next, we used M‐VCM to validate and found that overexpression of ATOX1 under radiation conditions significantly activated the AMPK signaling pathway (Figure 4H), effectively inhibited ROS generation (Figure 4I,L), stabilized mitochondrial membrane potential (Figure 4J,M), and promoted mPTP opening (Figure 4K,N). Knocking down AMPK significantly blocked these protective effects (Figure 4H–N), which was consistent with the previous cell experiment result. These results firmly substantiate that ATOX1 safeguards mitochondrial homeostasis and reins in oxidative stress in cardiomyocytes under radiation conditions by firing up the AMPK/NRF2 signaling pathway.

The regulatory mechanisms of ATOX1 in oxidative stress, mitochondrial homeostasis, and AMPK remain unclear. Further confirmation is needed to determine whether this is related to the function of ATOX1 as a copper chaperone protein and the regulatory pathway of the ATOX1‐DJ‐1‐AMPK axis.^11^, ^14^ Based on existing research findings, we proposed the following hypothesis: the regulatory mechanisms linking ATOX1 to oxidative stress, mitochondrial homeostasis, and the AMPK pathway remain poorly elucidated, which may be closely associated with the copper chaperone function of ATOX1 and the regulatory cascade of the ATOX1‐DJ‐1‐AMPK axis. To clarify whether ATOX1 exerts its biological functions through the ATOX1‐DJ‐1‐AMPK axis, three experimental groups were established in this study: the blank control group (oe‐NC + sh‐NC), the ATOX1 overexpression group (oe‐ATOX1+sh‐NC), and the ATOX1 overexpression combined with DJ‐1 knockdown group (oe‐ATOX1+sh‐DJ‐1). All cell groups were subjected to radiation intervention. WB results demonstrated that ATOX1 overexpression activated the AMPK signaling pathway under radiation conditions, and this activation effect was rescued by DJ‐1 knockdown (Figure S1A). IF assays further revealed that ATOX1 upregulation enhanced the fluorescence intensity of phosphorylated AMPK, whereas DJ‐1 silencing markedly reversed this trend (Figure S1B). Moreover, ATOX1 overexpression reduced mitochondrial ROS production, whereas DJ‐1 knockdown obviously increased ROS fluorescence signals (Figure S1C). Collectively, these results indicate that ATOX1 modulates oxidative stress in H9C2 cells by regulating the AMPK pathway in a DJ‐1‐dependent manner.

### 3.5 ATOX1 reduces myocardial injury and fibrosis in a murine thoracic irradiation model

**FIGURE 5:**
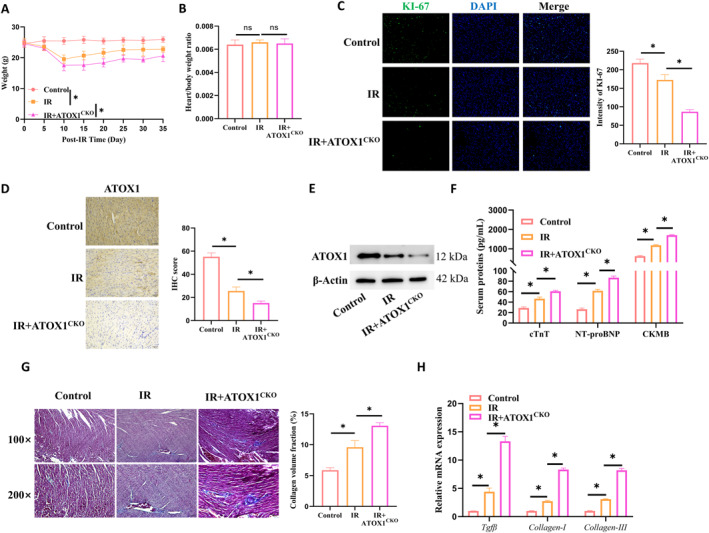
ATOX1 reduces myocardial injury and fibrosis in a murine thoracic irradiation model. (A) Post‐irradiation body weight changes in mice; (B) Heart‐to‐body weight ratio at the end of the experiment; (C) IF detection of KI‐67 in myocardial tissue; (D, E) IHC和 WB analysis of ATOX1 expression in myocardial tissue; (F) ELISA measurement of cTnT, NT‐proBNP, and CKMB in mouse serum; (G) Masson's trichrome staining to assess fibrosis in myocardial tissue; (H) RT‐PCR analysis of *Tgfβ*, *Collagen‐I*, and *Collagen‐III* mRNA levels in tissue. *p < 0.05.

We created a cardiac‐specific ATOX1 conditional knockout mouse model to examine the cardioprotective effects of ATOX1 in vivo. The experimental design included three groups: a blank control group (no radiation or knockout treatment), an IR group (radiation treatment without ATOX1 knockout), and an IR + ATOX1CKO group (radiation treatment combined with ATOX1 knockout). In the radiation‐treated groups, mice received targeted thoracic irradiation with lead shielding protecting the rest of their bodies. We tracked their body weight changes over time. Compared to the blank control group, both the IR group and the combined treatment group experienced a notable drop in body weight postradiation, which then gradually began to recover. The IR group showed a slightly quicker recovery, whereas the combined treatment group exhibited a more pronounced initial weight loss and had less weight recovery by the end of the experiment (Figure 5A). To calculate the heart‐to‐body weight ratio, the hearts were removed and weighed. The results showed that radiation treatment did not significantly affect this ratio (Figure 5B). IF was used to examine KI‐67 expression in myocardial tissue. The results demonstrated that radiation reduced KI‐67 expression, and this reduction was more pronounced after ATOX1 knockout (Figure 5C). Afterward, we evaluated the expression of ATOX1 in myocardial tissue. The results showed that radiation treatment materially decreased ATOX1 protein levels, with expression being even less evident after ATOX1 knockout (Figure 5D–E). Using ELISA, we analyzed indicators of myocardial damage in the serum of mice from all groups. The results revealed that after thoracic irradiation, the serum levels of cTnT, NT‐proBNP, and CKMB impressively rose, with the highest protein concentrations in the combined treatment group (Figure 5F). Masson staining was then used to assess the extent of fibrosis in cardiac tissue. Irradiation caused significant fibrotic changes in myocardial tissue, which were further worsened following ATOX1 knockout (Figure 5G). RT‐PCR was conducted to assess the mRNA expression levels of *Tgfβ*, *Collagen‐I*, and *Collagen‐III* in myocardial tissue. Radiation distinctly raised the mRNA levels of these genes, which matches the increase in heart damage markers in the serum. The combined treatment group had even higher mRNA levels (Figure 5H). Overall, our results point out that thoracic irradiation can cause palpable cardiac damage in vivo, but the expression of ATOX1 can partly safeguard the myocardium.

## 4 DISCUSSION

Our research study explores the mechanism by which ATOX1 protects against radiation‐induced myocardial damage via the AMPK/NRF2 pathway. Following radiation treatment, ATOX1 protein levels declined, leading to elevated mitochondrial ROS and mitochondrial dysfunction. These changes significantly weakened the proliferative capacity of cardiomyocytes. Overexpression of ATOX1 countered these effects by maintaining mitochondrial stability, reducing oxidative stress, and promoting cardiomyocyte proliferation and survival through the activation of the AMPK/NRF2 pathway. A thoracic radiation model was established in mice with cardiac‐specific ATOX1 knockout to study the effects of ATOX1 on radiation‐induced cardiac damage. In the irradiated group, the mice experienced weight loss, but the heart weight ratio remained relatively stable. The AMPK/NRF2 pathway was further suppressed. Following ATOX1 knockout, radiation‐induced myocardial fibrosis and injury were more pronounced. These results highlight the protective role of ATOX1 in mitigating radiation‐induced cardiac damage through the AMPK/NRF2 signaling pathway.

Radiation therapy has become one of the main forms of treating various types of cancer. This study used a single dose of 16–20 Gy to irradiate myocardial cells and mouse hearts, which is higher than the conventional clinical dose of 17 Gy^15^ and some animal models,^16^ but is not uncommon in RIHD studies. For example, multiple studies^17^, ^18^ have employed a similar single high‐dose irradiation regimen to rapidly induce injury and observe pathological changes. In this study, we observed that radiation exposure dramatically reduced the proliferative capacity of cardiomyocytes, boosted apoptosis, and downregulated ATOX1 expression. Moreover, ATOX1 knockout intensified radiation‐induced cardiac tissue damage and further increased serum levels of myocardial injury markers, such as cTnT, NT‐proBNP, and CKMB. ATOX1 encodes a copper chaperone that helps maintain copper homeostasis by binding copper ions in the cytoplasm and transporting them to the trans‐Golgi network.^19^ As studies have advanced, it has become evident that ATOX1 has multifaceted roles in cellular function. Not only does it facilitate copper ion transport but it also acts as an antioxidant. ATOX1 connects the Memo1 protein to the copper ion efflux pathway, protecting cells from copper‐induced damage.^20^ Moreover, ATOX1 triggers mitophagy, which helps maintain mitochondrial structure and reduces oxidative stress in hippocampal neurons.^11^ In the context of cellular health, mitophagy is a vital process. It selectively removes damaged mitochondria, reduces ROS production and maintains cellular homeostasis. In myocardial injury, a subtle activation of mitophagy acts as an adaptive mechanism for cardiomyocytes.^21^ In our research study, we noted that overexpressing ATOX1 preserved mitochondrial homeostasis in cardiomyocytes following radiation treatment, which led to a decrease in mitochondrial ROS. Based on these findings, we speculated that this protective mechanism may be closely related to the initiation of mitochondrial autophagy. However, we did observe that ATOX1 expression effectively activated the AMPK signaling pathway.

AMPK signaling is a critical pathway for mitophagy activation.^21^ The experiments we conducted revealed that radiation treatment suppressed AMPK activity, but overexpression of ATOX1 could counteract this suppression. Specifically, radiation reduced p‐AMPK levels, whereas ATOX1 overexpression increased AMPK phosphorylation and boosted the expression of downstream effectors such as NRF2 and HO‐1. The AMPK/NRF2/HO‐1 pathway, when activated, can both ramp up autophagy and reduce oxidative stress, thereby protecting cells.^22^, ^23^ NRF2, a transcription factor highly susceptible to oxidative stress, sees its activity nosedive after radiation exposure, with its nuclear translocation also markedly reduced.^24^ As Zhang et al.^25^ pointed out, phosphorylated AMPK can activate the downstream NRF2/HO‐1 signaling pathway, which reduces ROS in cardiomyocytes and robustly counteracts radiation‐induced myocardial oxidative stress and cardiac toxicity. Furthermore, the activation of the AMPK/NRF2 signaling pathway can effectively diminish doxorubicin‐induced ferroptosis in cardiomyocytes, which is reliant on mitochondrial function.^26^ Previous research studies have indicated that activating the AMPK/NRF2 pathway can enhance the expression of genes associated with mitochondrial fusion and endogenous antioxidants; this enhancement promotes mitochondrial fusion, optimizes mitochondrial dynamics and function, and mitigates oxidative stress damage in cardiomyocytes triggered by doxorubicin.^27^ In our study, we observed that radiation exposure dampened the activation of the AMPK/NRF2/HO‐1 axis, leading to a decline in the expression levels of related proteins, which suggests that radiation‐induced damage may exacerbate mitochondrial dysfunction and oxidative stress by suppressing this protective signaling pathway. Moreover, ATOX1 overexpression triggered a strong activation of the AMPK signaling pathway, which in turn reduced mitochondrial ROS levels significantly. AMPK activation also stabilized the mitochondrial membrane potential and limited the abnormal opening of mPTP, which is essential for maintaining mitochondrial function. In addition, cardiac fibrosis is one of the serious complications of radiation injury, which can lead to mechanical dysfunction and arrhythmia of the heart.^28^ This study confirms for the first time that knocking down ATOX1 exacerbates myocardial fibrosis lesions. Overall, it indicates that ATOX1 alleviates radiation‐induced mitochondrial dysfunction and oxidative stress damage in cardiomyocytes by activating the AMPK signaling axis, thereby improving cardiac injury.

In summary, our research study underscores the pivotal role of ATOX1 in safeguarding cardiomyocytes from radiation‐induced damage. By activating the AMPK/NRF2 signaling pathway, ATOX1 effectively combats oxidative stress and preserves mitochondrial integrity in cardiomyocytes. Although prior research studies have highlighted the significance of the AMPK/NRF2 axis in managing oxidative stress and mitochondrial homeostasis, the specific mechanisms through which ATOX1 enhances cardiomyocyte survival through this pathway have remained underexplored. Our study addresses this gap by offering a comprehensive analysis of the cardioprotective effects of ATOX1 under radiation stress, thus expanding our knowledge of its biological functions. Nonetheless, several limitations still exist. The occurrence of RIHD usually has a time lag, that is, the heart develops lesions after receiving radiotherapy for a long time. Considering that some studies typically have follow‐up periods of up to 6 months^29^ or even 18 months,^30^ we have not explored whether ATOX1 can exert long‐term cardioprotective effects. Therefore, in future research, the feasibility of ATOX1 as a potential cardiac protective target should be comprehensively evaluated by combining long‐term experiments and human follow‐up data to optimize radiotherapy regimens and prevent and manage cardiac complications in radiotherapy patients.

## AUTHOR CONTRIBUTIONS

**Wen Deng**: Conceptualization; methodology; data collection; investigation; data analysis; visualization; draft. **Li Su**: Data collection; data analysis; resources; investigation; project administration; revision.

## CONFLICT OF INTEREST STATEMENT

The authors declare no conflicts of interest.

## ETHICS STATEMENT

Experiments were approved by the ethics committee of Guangdong Medical Laboratory Animal Center, approval number D202506‐11, in compliance with institutional guidelines for the care and use of animals.

## Supporting information

Figure S1

## Data Availability

All the data within this manuscript could be gotten from corresponding author upon reasonable request.
