# Supplementary material for: ATOX1 alleviates radiation‐induced cardiac injury by modulating AMPK/NRF2 to inhibit myocardial oxidative stress and mitochondrial dysfunction
Source: J Cell Commun Signal. 2026 May 15;20(2):e70079. doi: 10.1002/ccs3.70079 (PMC13179142; doi:10.1002/ccs3.70079)
Supplement: Supplementary file 1 — Figure S1 [file CCS3-20-e70079-s001.docx]

**
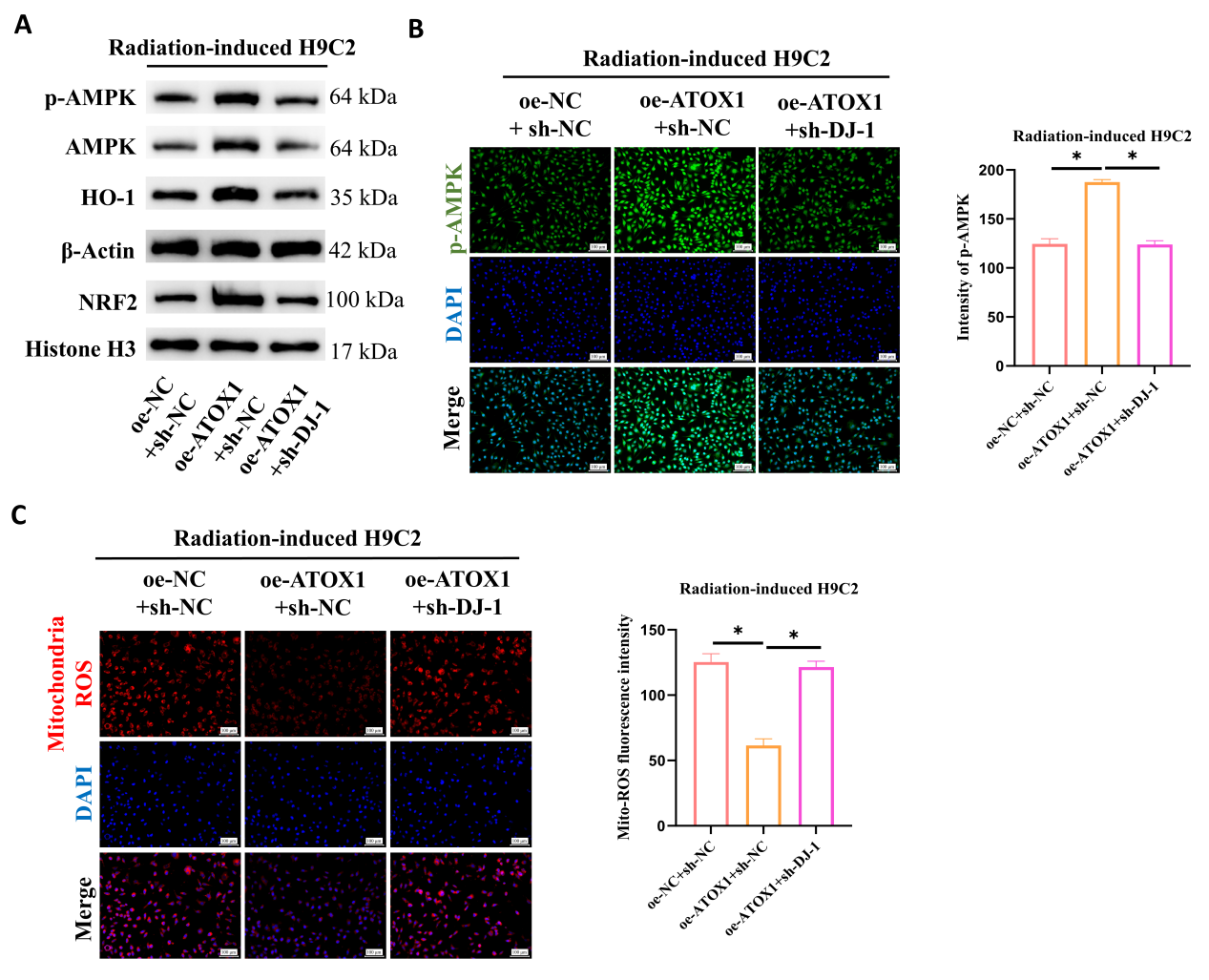
**

**Figure S1**

A: WB assessed the activation of the AMPK/NRF2 signaling pathway; B: IF detection of AMPK phosphorylation levels in different cell groups; C: Assay kit measured and quantified mitochondrial ROS levels in H9C2 cells. **P* < 0.05.
